# Free Volume in Membranes: Viscosity or Tension?

**DOI:** 10.4236/ojbiphy.2015.53007

**Published:** 2015-07-22

**Authors:** V. S. Markin, F. Sachs

**Affiliations:** 1 Department of Anesthesiology and Pain Management, UT Southwestern, Dallas, TX, USA; 2 Physiology & Biophysical Sciences, SUNY Buffalo, Buffalo, NY, USA

**Keywords:** Fluorescence, Polarization, Anisotropy, Lipid, Bilayer

## Abstract

Many papers have used fluorescent probe diffusion to infer membrane viscosity but the measurement is actually an assay of the free volume of the membrane. The free volume is also related to the membrane tension. Thus, changes in probe mobility refer equally well to changes in membrane tension. In complicated structures like cell membranes, it appears more intuitive to consider variations in free volume as referring to the effect of domains structures and interactions with the cytoskeleton than changes in viscosity since tension is a state variable and viscosity is not.

Many papers have used fluorescent probe polarization or anisotropy to infer membrane viscosity [1]–[8] and a recent paper has referred to the effect of gravity on membrane viscosity [9]. However, the mobility of membrane soluble probes is really an assay of the free volume of the membrane, and the free volume is directly related to the tension in the membrane, and as a state variable tension is a better option to describe the effect of perturbations. It is a lot simpler to understand how gravity could affect the tension in a liposome resting on a substrate than its viscosity. Changes in probe mobility, especially in complex heterogeneous biological membranes, can easily because by changes in the line tension of domains and interactions with the cytoskeleton. If we consider mechanosensitive channels, it is much simpler to create a model of the stretch sensitivity [10] using membrane tension [11] [12] than by using membrane viscosity. Since nearly all papers using fluorescent probe mobility to study membrane properties refer to measurements of membrane viscosity, we thought it would be useful to show how those measurements can be interpreted in other ways.

There is an extensive literature on liquid viscosity and numerous theories have been based on the assumption of a quasicrystalline structure. A molecule is pictured as vibrating about an equilibrium position until the combination of two events occurs: (1) the molecule attains sufficient energy to overcome the attractive forces holding it to its neighbors, and (2) an empty site is available into which the molecule can jump [13]. This developed into the free volume approach to liquid viscosity. Doolittle [14] and later Williams, Landel and Ferry [15] showed that the viscosity η can be related to the free volume of the liquid *f* by the relation

(1) η=Aexp(B∕f)

where *A* and *B* are constants, the latter being about unity. This approach successfully explained the data on temperature and pressure dependence of liquid viscosity. However, the free volume of lipid membranes is directly related to membrane tension so measures of probe mobility also reflect membrane tension [16].

In case of a membrane, the role of pressure in bulk liquids is played by membrane tension γ [17], more precisely, the surface pressure π = γ_0_ − γ. Increased tension increases membrane area and free volume, and hence to a decrease in viscosity. If we consider the membrane as a square latticework of lipids with unit area a, stretching the membrane will change the unit area to *a* + Δ*a*. This change of unit area is related to tension by the area elasticity, *k_a_*, which is the reciprocal of the elasticity E,

(2) $$\frac{\Delta A}{a}=\Delta \gamma ∕E.$$.

One could assume that the free volume (free area) of a lipid molecule in a membrane is only a fraction ρ of the unit area, and this fraction is close to unity. Then the free area can be presented as:

(3) $$f=\rho (a+\Delta a)=\rho (a+k_{a}\;\gamma )$$

and the viscosity can be presented as:

(4) $$\eta =A\;\exp\left[\frac{B}{\rho (a+k_{a}\;\gamma )}\right]$$

The effect of viscosity on probe polarization P is typically evaluated in bulk solutions such as glycerol, so we have available a known function *P*(η). A change of polarization Δ*P* that suggests an apparent change of viscosity of Δη, is equivalent to a change of tension of,

(5) Δγ=−ΔηB∕ρ(γka+a)

Thus, a change in apparent viscosity is proportional to a change in tension so the two interpretations cannot be distinguished from the probes.

Biological membranes have gradients in free volume, or equivalently, viscosity and tension, due to the presence of domains with line tension and changes in composition [18] and by the sharing of stress between the cytoskeleton and the bilayer [19]. The mechanosensitive channels Piezo1 [20]–[23] and TREK-1 [24] and MscL [25]–[27] have very similar responses to membrane tension, but, if they are segregated into separate domains and hence are subjected to different tensions. The observed dose-response curves of these channels, Popen vs mean applied tension, could be different [28], and the results may be misinterpreted as differences in protein dynamics [11] [29].

To think of the effects of domains on the data, visualize the membrane as a continuous layer (*C*) containing disk shaped domains (*D*). These two parts may have different mechanical properties: surface tension (γ_*C*_ and γ_*D*_), free area (*f_C_* and *f_D_*) and viscosity (η_*C*_ and η_*D*_). Surface tension (η_*C*_ and η_*D*_) can be related to the mean radius of the domain r and the line tension α:

(6) $$\gamma_{C}-\gamma_{D}=\alpha ∕r.$$.

If two mechanosensitive channels (like Piezo and TREK), find themselves in separate domains, say the continuous phase *C* and domain *D*, their dose response curves P1(γ) and P2(γ) will be shifted along the axes of tension by the amount of α/*r*. There are many reports of spatial domains with different viscosity that are equally well interpreted as domains of different tension. Thermodynamics summarizes gradient of free energy in crossing the domain boundary as the line tension. The line tension is related to free volume, or equivalently, elasticity and viscosity.

Since tension is a state variable it can be measured in other ways than probe mobility. All of the literature on mechanosensitive channels use tension as the controlling variable and that is measured commonly by the application of Laplace's law that relates the tension in a constrained membrane to the hydrostatic pressure across the membrane and it curvature. Viscosity does not enter the calculation so tension can be measured separately from viscosity. Measurements using probe mobility to infer viscosity should consider performing macroscopic mechanics [10] [12] studies to separate tension effects from viscosity effects.
